# *In vitro* antiplasmodial, antileishmanial and antitrypanosomal activities of selected medicinal plants used in the traditional Arabian Peninsular region

**DOI:** 10.1186/1472-6882-12-49

**Published:** 2012-04-20

**Authors:** Nawal M Al-Musayeib, Ramzi A Mothana, An Matheeussen, Paul Cos, Louis Maes

**Affiliations:** 1 Department of Pharmacognosy, College of Pharmacy, King Saud University, P.O. Box 2457, Riyadh, 11451, Saudi Arabia; 2 Laboratory for Microbiology, Parasitology and Hygiene (LMPH), Faculty of Pharmaceutical, Biomedical and Veterinary Sciences, Antwerp University, Universiteitsplein 1, 2610, Wilrijk-Antwerp, Belgium

## Abstract

**Background:**

Worldwide particularly in developing countries, a large proportion of the population is at risk for tropical parasitic diseases. Several medicinal plants are still used traditionally against protozoal infections in Yemen and Saudi Arabia. Thus the present study investigated the *in vitro* antiprotozoal activity of twenty-five plants collected from the Arabian Peninsula.

**Methods:**

Plant materials were extracted with methanol and screened *in vitro* against erythrocytic schizonts of *Plasmodium falciparum*, intracellular amastigotes of *Leishmania infantum* and *Trypanosoma cruzi* and free trypomastigotes of *T. brucei*. Cytotoxic activity was determined against MRC-5 cells to assess selectivity. The criterion for activity was an IC_50_ < 10 μg/ml (<5 μg/ml for *T. brucei*) and selectivity index of >4.

**Results:**

Antiplasmodial activity was found in the extracts of *Chrozophora oblongifolia*, *Ficus ingens*, *Lavandula dentata* and *Plectranthus barbatus*. Amastigotes of *T. cruzi* were affected by *Grewia erythraea*, *L. dentata*, *Tagetes minuta* and *Vernonia leopoldii*. Activity against *T. brucei* was obtained in *G. erythraea*, *L. dentata*, *P. barbatus* and *T. minuta*. No relevant activity was found against *L. infantum*. High levels of cytotoxicity (MRC-5 IC_50_ < 10 μg/ml) and hence non-specific activities were noted in *Cupressus sempervirens*, *Kanahia laniflora* and *Kniphofia sumarae*.

**Conclusion:**

The results endorse that medicinal plants can be promising sources of natural products with antiprotozoal activity potential. The results support to some extent the traditional uses of some plants for the treatment of parasitic protozoal diseases.

## Background

Today over one billion people worldwide are at risk for tropical diseases caused by parasitic organisms. The World Health Organization (WHO) now classifies many as neglected tropical diseases, having an enormous impact on socioeconomic development and quality of life at all levels particularly in developing countries [1]. At present, a lot of research is committed to leishmaniasis, malaria, Chagas disease and sleeping sickness, not only because they are major killing diseases but also because disease control becomes more difficult due to a number of factors that limit the utility of current drugs in resource-poor settings, such as high cost, poor compliance, drug resistance, low efficacy and poor safety [2]. Hence, the search for new and preferably cheap drugs needs to be continued [3].

Natural products are still major potential sources of innovative therapeutic agents for various conditions, including infectious diseases as they represent an unmet source of chemical diversity [4]. Indeed, several antiparasitic drugs have been derived directly from natural sources, such as quinine, artemisinin and atovaquone as antimalarials and amphotericin B as antileishmanial drug.

It is estimated that two thirds of the world population still rely on traditional medical remedies, mainly plants, because of limited availability and affordability of pharmaceutical medicines [5]. This explains why a lot of current research focuses on natural molecules and plant-derived products as they can be sourced easily, are locally available and can be selected on the basis of their ethnomedicinal use [6].

In this study, 25 plants were selected from the flora of Yemen and Saudi Arabia, and subjected to a broad panel of *in vitro* antiparasitic assays in an attempt to identify plant species with a promising antiprotozoal *in vitro* activity profile and could be subject for further investigations.

## Methods

### Plant materials

Twenty five plants (Table 1) were selected randomly from different areas of Yemen and Saudi Arabia in March and Jun 2008 and identified at the Pharmacognosy Departments, Colleges of Pharmacy, King Saud and Sana'a Universities, Saudi Arabia and Yemen. The plants were selected mainly on the basis of their local medicinal knowledge. Voucher specimens were deposited at departments. The botanical names, plant part used and the traditional uses of the plants in the collected areas are presented in Table 1.

**Table 1:** List of plants screened and their traditional uses

**Plant species**	**Voucher specimen no.**	**Family**	**Part used**	**Traditional uses**
*Ajuga bracteosa* Wall. ex Benth.	Mo-I10a	Labiatae	L, F	As antiseptic and for teeth pains, stimulant, diuretic and in treatment of rheumatism, gout, palsy, amenorrhea and malaria ^a,b^
*Caralluma quadrangula* (Forssk.) N.E.Br.	Mo-H02a	Asclepiadaceae	L	For diabetes, stomachic ulcer and smallpox ^a, c^
*Centaurea pseudosinaica* Czerep.	Mo-S11a	Asteraceae	L, T	For wounds and kidney diseases ^a^
*Chrozophora oblongifolia* (Del.) A. Juss. ex Spreng.	Mo-S02a	Euphorbiacea	L, S	As antiseptic for wounds, antimicrobial, cathartic, emetic and hypoglycemic and for hemorrhoids ^a, d^
*Costus arabicus* L.	Mo-S05a	Zingiberaceae	R	For cancers ^a^
*Cupressus sempervirens* L.	Mo-S25a	Cupressaceae	L	As expectorant, astringent and for wounds, diarrhea, hemorrhoids ^a, c^
*Dodonaea viscosa* (L.) Jacq.	Mo-T01a	Sapindaceae	L, S	For malaria, wounds and burns, gout , rheumatism and as anesthetic, laxative and tonic ^a, c, e, f,^
*Dorstenia barnimiana* Schweinf.	Mo-T09	Moraceae	L, S	For the treatment of fungal and skin diseases ^a, c^
*Enicostemma verticillare* (Retz.) Baill.	Mo-I06a	Gentianaceae	L	For diabetes ^a^
*Ficus cordata ssp. Salicifolia*	15133	Moraceae	L, S	For the treatment of filariasis, diarrheal infections, tuberculosis and oral infections ^a, c^
*Ficus ingens* (Miq.)	15187	Moraceae	L, S	For Piles, diarrhea, and as diuretic ^a^
*Ficus palmata* Forssk	15163	Moraceae	L, S	For constipation and lungs and bladder diseases ^a, c^
*Grewia erythraea* Schweinf.	Mo-S07a	Tiliaceae	L, S	As Diuretic and haemostatic and for kidney diseases ^a^
*Iris albicans* Lange	Mo-I02a	Iridaceae	R	For rheumatism and gout ^a^
*Kanahia laniflora* (Forsk.) R. Br.	Mo-I19a	Asclepiadaceae	L, T	For tumors, skin diseases, scabies and itching ^a, c, h^
*Kniphofia sumarae* Deflers	Mo-I10a	Liliaceae	R	For malaria ^a^
*Lavandula dentata* L.	Mo-I11a	Labiatae	L, F	For wounds, rheumatism, urine retention, and kidney stones and as antiseptic ^a, d^
*Leucas inflata* Benth.	Mo-I05a	Labiatae	L, F	For kidney diseases and tooth ache ^a^
*Pulicaria inuloides* DC.	Mo-M05a	Asteraceae	L, F	For wounds and as antiseptic ^a^
*Plectranthus barbatus* Andr.	15732a	Labiatae	L, S	For stomachache, nausea, gastritis, intestinal spasms, burns, wounds, sores, insect bites, allergies, ringworms, infections, malaria and break fevers ^a, f^
*Rhus retinorrhaea* Steud. ex Oliv.	Mo-T22a	Anacardiaceae	L	General tonic and for painful joints ^a^
*Tagetes minuta* L.	YT-20a	Asteraceae	L, S	As antimicrobial, anthelmintic, diuretic, and antispasmodic agent ^a, f^
*Tarconanthus camphoratus* L	Mo-S15a	Asteraceae	L, T	For wounds and for urinary tract infections ^a^
*Teucrium yemense* Deflers	Mo-S17a	Labiatae	L, F	For kidney diseases, rheumatism and diabetes ^a, d^
*Vernonia leopoldii* Vatke	Mo-T16a	Asteraceae	L, F	For cough, colic and skin diseases ^a, h^

*F* Flower, *L* Leaves, *R* Roots or rhizomes, *S* Stems, *T* Fruits.

^a^ information has been taken from native people.

^b^ Chandel S, Bagai U, 2010 [7].

^c^ Al-Dubai and Al-Khulaidi (1996) [8].

^d^ Atiqur-Rahman et al., (2004) [9].

^e^ Mossa et al., (1987) [10].

^f^ Ali et al., (2004) [11].

^g^ Fleurentin and Pelt (1982) [12].

^h^ Schopen (1983) [13].

### Extraction of plant materials

The air-dried and powdered plant material (50 g) was extracted with 500 ml methanol (CH_3_OH) by using a Soxhlet apparatus for 8 hours. The obtained methanol extract was filtered and evaporated by using a rotatory evaporator and freeze dryer. The dried extracts were stored at −20°C until used. Stock solutions were prepared in 100% DMSO at 20 mg/ml just prior to screening.

### Reference drugs

For the different tests, appropriate reference drugs were used as positive control: tamoxifen for MRC-5, chloroquine for *P. falciparum*, miltefosine for *L. infantum*, benznidazole for *T. cruzi* and suramin for *T. b. brucei*. All reference drugs were either obtained from the fine chemical supplier Sigma or from WHO-TDR.

### Biological assays

The integrated panel of microbial screens and standard screening methodologies were adopted as previously described [14]. All assays were performed in triplicate (first test in duplicate and a single independent repeat) at the Laboratory of Microbiology, Parasitology and Hygiene at the University of Antwerp, Belgium. Plant extracts were tested at 5 concentrations (64, 16, 4, 1 and 0.25 μg/ml) to establish a full dose-titration and determination of the IC_50_ (inhibitory concentration 50%). The concentration of DMSO did not exceed 0.5%. The selectivity of action was assessed by simultaneous evaluation of cytotoxicity on a fibroblast (MRC-5) cell line. The criterion for activity was an IC_50_ < 10 μg/ml (< 5 μg/ml for *T. brucei*) and a selectivity index of > 4.

### Antileishmanial activity

*L.infantum* MHOM/MA(BE)/67 amastigotes were collected from the spleen of an infected donor hamster and used to infect primary peritoneal mouse macrophages. To determine *in vitro* antileishmanial activity, 3 × 10^4^ macrophages were seeded in each well of a 96-well plate. After 2 days outgrowth, 5 × 10^5^ amastigotes/well were added and incubated for 2 h at 37°C. Pre-diluted plant extracts were subsequently added and the plates were further incubated for 5 days at 37°C and 5% CO_2_. Parasite burdens (mean number of amastigotes/macrophage) were microscopically assessed after Giemsa staining, and expressed as a percentage of the blank controls without plant extract.

### Antiplasmodial activity

Chloroquine-resistant *P. falciparum* 2/K 1-strain was cultured in human erythrocytes O^+^ at 37°C under a low oxygen atmosphere (3% O_2_, 4% CO_2_, and 93% N_2_) in RPMI-1640, supplemented with 10% human serum. Infected human red blood cells (200 μl, 1% parasitaemia, 2% haematocrit) were added to each well and incubated for 72 h. After incubation, test plates were frozen at −20°C. Parasite multiplication was measured by the Malstat method [14,15].

### Antitrypanosomal activity

*Trypanosoma brucei* Squib-427 strain (suramin-sensitive) was cultured at 37°C and 5% CO_2_ in Hirumi-9 medium [16], supplemented with 10% fetal calf serum (FCS). About 1.5 × 10^4^ trypomastigotes/well were added to each well and parasite growth was assessed after 72 h at 37°C by adding resazurin [17]. For Chagas disease, *T. cruzi* Tulahuen CL2 (benznidazole-sensitive) was maintained on MRC-5 cells in minimal essential medium (MEM) supplemented with 20 mM L-glutamine, 16.5 mM sodium hydrogen carbonate and 5% FCS. In the assay, 4 × 10^3^ MRC-5 cells and 4 × 10^4^ parasites were added to each well and after incubation at 37°C for 7 days, parasite growth was assessed by adding the β-galactosidase substrate chlorophenol red β-D-galactopyranoside [18]. The color reaction was read at 540 nm after 4 h and absorbance values were expressed as a percentage of the blank controls.

#### Cytotoxicity assay

MRC-5 SV2 cells were cultivated in MEM, supplemented with L-glutamine (20 mM), 16.5 mM sodium hydrogen carbonate and 5% FCS. For the assay, 10^4^ MRC-5 cells/well were seeded onto the test plates containing the pre-diluted sample and incubated at 37°C and 5% CO_2_ for 72 h. Cell viability was assessed fluorimetrically after 4 hours of addition of resazurin. Fluorescence was measured (excitation 550 nm, emission 590 nm) and the results were expressed as % reduction in cell viability compared to control.

## Results

Crude methanol extracts from 25 plant species belonging to 18 families that are used in Arabian traditional medicine, were evaluated in the integrated *in vitro* screen for antileishmanial, antiplasmodial and antitrypanosomal potential (Table 2). Only 7 extracts exhibited relevant activity (acceptable potency and selectivity) in one or more models (Table 2).

**Table 2:** Antiprotozoal activity of the methanol extracts of the investigated plants and their cytotoxicity against MRC-5 cell lines

**Plant species**	***P. falciparum***		***L. infantum***		***T. cruzi***		***T. brucei***		***MRC-5***
	**IC**_**50**_	**SI**	**IC**_**50**_	**SI**	**IC**_**50**_ **(μg/ml)**	**SI**	**IC**_**50**_	**SI**	**IC**_**50**_
*Ajuga bracteosa*	>64.0	>1	>64.0	>1	28.8 ± 4.6	>2.22	31.2 ± 5.2	>2.05	>64.0
*Caralluma quadrangula*	27.5 ± 4.3	>2.33	>64.0	>1	>64.0	>1	32.5 ± 6.5	>1.97	>64.0
*Centaurea pseudosinaica*	48.2 ± 9.8		32.5 ± 3.5		31.0 ± 6.1		9.1 ± 1.8	1.76	16.0 ± 5.3
*Chrozophora oblongifolia*	5.0 ± 1.2	12.80	27.3 ± 2.8	2.34	32.0 ± 5.8	>2	10.8 ± 2.4	5.93	>64.0
*Costus arabicus*	>64.0		27.3 ± 3.1	1.41	13.8 ± 2.1	2.79	30.0 ± 4.9	1.28	38.5 ± 8.2
*Cupressus sempervirens*	7.6 ± 2.4	1.41	2.0 ± 0.4	5.35	8.3 ± 1.9	1.29	2.1 ± 0.2	5.10	10.7 ± 3.1
*Dorstenia barnimiana*	34.2 ± 8.7	1.44	>64.0		29.6 ± 3.9	1.67	22.6 ± 5.8	2.19	49.4 ± 9.1
*Dodonaea viscosa*	46.7 ± 11.8	>1.37	45.3 ± 11.8	1.41	>64.0	>1	11.1 ± 1.8	5.77	>64.0
*Enicostemma verticillare*	>64.0 ±	>1	>64.0	>1	>64.0	>1	9.9 ± 1.1	>6.46	>64.0
*Ficus cordata ssp.salicifolia*	27.0 ± 6.9	1.20	27.3 ± 6.1	1.19	26.3 ± 3.2	1.24	8.2 ± 1.9	3.96	32.5 ± 7.3
*Ficus ingens*	8.4 ± 2.3	3.87	32.5 ± 7.2	1.00	31.2 ± 4.3	1.04	8.0 ± 2.2	4.06	32.5 ± 7.5
*Ficus palmata*	14.5 ± 3.8	2.60	>64.0		22.6 ± 4.0	1.67	8.1 ± 2.6	4.65	37.7 ± 6.9
*Grewia erythraea*	11.7 ± 3.5	2.32	24.1 ± 3.8	1.13	8.2 ± 1.8	3.32	2.6 ± 0.9	10.46	27.2 ± 6.1
*Iris albicans*	55.5 ± 6.2	>1.15	>64.0	>1	>64.0	>1	10.6 ± 3.1	>6.04	>64.0
*Kanahia laniflora*	27.9 ± 4.9		>64.0		0.4 ± 0.2	2.00	9.6 ± 3.0		0.8 ± 0.2
*Kniphofia sumarae*	1.3 ± 0.6	5.69	32.5 ± 4.9		31.4 ± 3.4		5.9 ± 2.8	1.25	7.4 ± 1.4
*Lavandula dentata*	7.1 ± 1.4	4.17	20.3 ± 3.5	1.46	7.9 ± 0.5	3.75	3.0 ± 1.8	9.87	29.6 ± 5.9
*Leucas inflata*	44.6 ± 6.3		>64.0		>64.0		8.4 ± 2.2	3.51	29.5 ± 6.0
*Plectranthus barbatus*	6.5 ± 2.0	5.06	24.1 ± 2.9	1.37	23.3 ± 2.9	1.41	2.6 ± 1.8	12.65	32.9 ± 6.8
*Pulicaria inuloides*	21.6 ± 3.8	>2.96	45.3 ± 8.3	1.41	31.7 ± 4.0	>2.02	7.8 ± 2.1	>8.21	>64.0
*Rhus retinorrhaea*	37.1 ± 4.9	1.43	>64.0		30.5 ± 3.9	1.74	34.0 ± 5.8	1.56	53.2 ± 7.2
*Tagetes minuta*	14.0 ± 2.8	4.57	30.1 ± 4.6	>2.13	9.2 ± 1.9	>6.96	2.2 ± 1.5	>29.09	>64.0
*Tarconanthus camphoratus*	>64.0	>1	>64.0	>1	>64.0	>1	>64.0	>1	>64.0
*Teucrium yemense*	12.5 ± 2.6	2.18	32.5 ± 6.6		30.5 ± 2.9		7.1 ± 2.3	3.83	27.2 ± 5.3
*Vernonia leopoldii*	41.9 ± 7.9		27.3 ± 5.1	1.10	9.2 ± 1.2	3.27	8.0 ± 2.9	3.76	30.1 ± 4.9
Chloroquine	0.3 ± 0.1		-		-		-		-
Miltefosine	-		3.32 ± 0.7		-		-		-
Benznidazole	-		-		2.2 ± 0.5		-		-
Suramin	-		-		-		0.03 ± 0.02		-
Tamoxifen	-		-		-		-		11.0 ± 2.3

IC_50_ values of reference drugs are expressed in μM/ml concentrations.

### Antimalarial activity

In this study, the methanol extract of *Chrozophora oblongifolia* exhibited the greatest activity against *P. falciparum* with an IC_50_ value of 5.0 μg/ml and a high SI value of 12.8. Furthermore, the extract of three other plants (*Ficus ingens*, *Lavandula dentata* and *Plectranthus barbatus*) showed activity against *P. falciparum* with IC_50_ 8.4, 7.1 and 6.5 μg/ml respectively. These extracts exhibited moderate SI values of 3.8, 4.1 and 5.1, respectively.

### Antileishmanial activity

No relevant results were found against *L. infantum*. A very marginal activity was observed for *C. oblongifolia*, *Costus arabicus*, *Grewia erythraea*, *L. dentata*, *P. barbatus*, and *Vernonia leopoldii* with IC_50_ values between 20.3 and 27.3 μg/ml and low SI values between 1.0 and 2.5.

### Antitrypanosomal activity

Our screen demonstrated that *T. b. brucei* is more sensitive than *T. cruzi* towards the investigated plant extracts (Table 2). The results revealed that the extract of *G. erythraea* showed activity against *T. cruzi* (IC_50_ 8.2 μg/ml) and *T. brucei* (IC_50_ 2.6 μg/ml). Additionally, *L. dentata* demonstrated activity against *T. cruzi* and *T. brucei* with IC_50_ values of 7.9 and 3.0 μg/ml respectively. Meanwhile, the extract of *Tagetes minuta* showed less activity against *T. cruzi* with an IC_50_ value of 9.2 (SI = 6.9) and higher activity against *T. brucei* with an IC_50_ value of 2.2 μg/ml and the highest SI value of >29.1. On the other hand, the methanol extract of *P. barbatus*, showed antitrypanosomal activity only against *T. brucei* (IC_50_ 2.6 μg/ml) with high SI value of 12.6, while the extract of *V. leopoldii* showed activity against both *T. cruzi* and *T. brucei* (IC_50_ 9.2 and 8.0 μg/ml) with low SI values of 3.2 and 3.7 respectively*.*

### Cytotoxicity assay

The highest cytotoxic effect against MRC-5 cells was obtained with the methanol extract of *Kanahia laniflora* (IC_50_ of 0.83 μg/ml). The extracts of *Kniphofia sumarae* and *Cupressus sempervirens* also exhibited a noticeable cytotoxic effect with IC_50_ values of 7.7 and 10.7 μg/ml respectively (Table 2).

## Discussion

The scientific evaluation of medicinal plants used in the preparation of folk remedies has provided modern medicine with several effective pharmaceuticals for the treatment of diseases caused by protozoan parasites [19,20]. As a result of this, during the last two decades numerous studies from various parts of the world on antiprotozoal activity of medicinal plants have been reported [21-26].

In continuation of our search for substances of plant origin with pharmacological effects, we have screened 25 plants collected from Saudi Arabia and Yemen for their antiplasmodial, antileishmanial and antitrypanosomal activities. It is important to mention that at the best of our knowledge, this study represents the first report on antiprotozoal activities for most part of the investigated plants. Although few plants are partly investigated, existing knowledge remains in many cases very limited. Based on the activity (IC_50_) and selectivity, seven plant extracts could be considered as promising and interesting enough to engage in further purification and evaluation.

During the course of screening, it was found that the methanol extract of the *C. oblongifolia*, collected from Yemen, exhibited the greatest antiplasmodial activity. Our result is in agreement with data reported recently by Abdel-Sattar et al. (2010) [27], which showed antiplasmodial activity for this species collected from Saudi Arabia (IC_50_ 4.8 μg/ml) with a better selectivity (SI > 13.2). Moreover, Benoit-Vical et al. (2008) [28] reported that the water extract of *Chrozophora senegalensis* showed a remarkable *in vitro* antimalarial activity (IC_50_ 1.6 μg/ml).

Other interesting and promising source of antiplasmodial and antitrypanosomal activities is *F. ingens*. It is worth to mention that this is the first report on the antiprotozoal activity of *F. ingens*. Other *Ficus* species like *F. citrifoliaF. fistulosa*, and *F. sur* demonstrated a noticeable antimalarial activity [29-31]. Moreover, bioassay-guided fractionation of the extract from the dried leaves and stem barks of *F. fistulosa* led to the isolation of trichothecene sesquiterpenoids, including verrucarin L acetate which was found to inhibit the growth of *P. falciparum* with IC_50_ values below 1 ng/ml [30].

Another interesting plant was *G. erythraea*, which demonstrated considerable antimalarial and antitrypanosomal activities. Our data are in agreement with literature data of other *Grewia* species such as *G. hexaminta* and *G. bilamellata*[32,33]. Ma et al., (2006) [32] demonstrated that some triterpenoids e.g. 3α,20-lupandiol, grewin, nitidanin and 2α,3β-dihydroxy-olean-12-en-28-oic acid isolated from *G. bilamellata* are responsible for the antimalarial effect and showed varying degrees of *in vitro* activity against *P. falciparum*. The presence of such terpenoids in our *G. erythraea* may explain the biological effects seen in our screen.

Moreover, one of the most interesting plants was *L. dentata* collected from Yemen. Our antiparasitic screening revealed remarkable *in vitro* antiplasmodial and antitrypanosomal activity observed for *L. dentata* but with moderate SI of 4.1 and 9.8. These findings are in agreement with literature data published recently by Abdel-Sattar et al. (2010) [27] who reported the antiparasitic activity of the methanol extract of *L. dentata* growing in Saudi Arabia. The extract of *L. dentata* growing in Saudi Arabia showed better selectivity for *P. falciparum* (SI = 32.1) as compared with our results. This can be attributed to variation in the area of collection and ecological factors, which has a great impact on the quality and quantity of plants constituents. Apparently the activity of this species is mostly attributed to the presence of essential oil which was revealed to be responsible for antiparasitic and antibacterial activities [34,35].

Tempone et al., (2008) [36] investigated the antileishmanial activity of some Brazilian flora extracts, including *P. barbatus* which showed activity against *L. chagasi* with EC_50_ value of 54.5 μg/ml. In earlier studies, several *Plectranthus* species showed antiplasmodial activity against *P. falciparum* 3D7 strain [37,38]. The results obtained in the present screen are in agreement with the literature data found and hence justifies the folkloric use. In addition to that, Van Zyl et al., (2008) [38] attributed the antiplasmodial activity of these *Plectranthus* species to the presence of abietane diterpenes.

Whereas the crude extract of *T. minuta* showed a remarkable antitrypanosomal activity against both trypanosome species, no effect was found against *P. falciparum*. Obviously our results of the antiplasmodial activity of *T. minuta* were not in agreement with the antimalarial effect noted recently by Lacroix et al., (2011) and Shahzadi et al., (2010) [39,40]. It was demonstrated that the ethyl acetate as well as *n*-hexane extract exhibited a notable antimalarial activity at 2.78 μg/ml against *P. falciparum* 3D7 strain. Apparently these findings are attributed to the presence of essential oil as well as sesquiterpene lactones.

In our screen *A. bracteosa* didn't show any interesting antiprotozoal activity. These results are not in agreement with those recently reported by Chandel and Bagai (2010) [7]. In contrast to several reports on *Vernonia* species e.g. *V. amygdalinaV. brachycalyx.V. cinerea* and *V. colorata* indicating *in vitro* and *in vivo* antiplasmodial activity [41-43], our extract of *V. leopoldii* showed no antiplsmodial activity. On the other hand, *V. leopoldii* showed a notable antitrypanosomal activity, which was in agreement with the results obtained by Hoet et al. (2004) [44] who attributed the antitrypanosomal activity to the presence of stigmastane-type steroids e.g. vernoguinosterol and vernoguinoside, which were isolated from the stem bark of *V. guineensis*. Such compounds could also be responsible for the observed effect of *V. leopoldii*.

## Conclusion

In conclusion, the results show that scientific studies carried out on medicinal plants having traditional claims of effectiveness can yield fruitful results. The present work led to the identification of seven plant extracts exhibiting relevant antiprotozoal potential namely *C. oblongifolia*, *F. ingens*, *G. erythraea*, *L. dentata*, *P. barbatus*, *T. minuta* and *V. leopoldii*. Moreover, the results in the present study support to some extent the traditional uses of some plants for the treatment of parasitic diseases. Studies aimed at the isolation and structure elucidation of antiprotozoal active constituents from some investigated plants are now in progress.

## Competing interests

The author(s) declare that they have no competing interests.

## Authors' contributions

RAM and NMA carried out the study design, plant collection and extraction, part of the experimental work, data collection and interpretation, literature search and manuscript preparation. AM carried out the *in vitro* assays, PC and LM evaluated the data and corrected the manuscript for publication. All authors read and approved the final manuscript.

## Pre-publication history

The pre-publication history for this paper can be accessed here:

http://www.biomedcentral.com/1472-6882/12/49/prepub
